# Establishing post mortem criteria for the metabolic syndrome: an autopsy based cross-sectional study

**DOI:** 10.1186/s13098-018-0339-0

**Published:** 2018-04-25

**Authors:** Martin Roest Christensen, Anne Bugge, Mariam Elmegaard Malik, Jørgen Lange Thomsen, Niels Lynnerup, Jørgen Rungby, Jytte Banner

**Affiliations:** 1 Department of Forensic Medicine, University of Copenhagen, Copenhagen, Denmark; 2 Department of Forensic Medicine, University of Southern Denmark, Odense, Denmark; 3 Department of Endocrinology, Bispebjerg Hospital, Copenhagen, Denmark

**Keywords:** Metabolic syndrome, Autopsy, Post mortem application, Severe mental illness, SURVIVE study

## Abstract

**Background:**

Individuals who suffer from mental illness are more prone to obesity and related co-morbidities, including the metabolic syndrome. Autopsies provide an outstanding platform for the macroscopic, microscopic and molecular-biological investigation of diseases. Autopsy-based findings may assist in the investigation of the metabolic syndrome. To utilise the vast information that an autopsy encompasses to elucidate the pathophysiology behind the syndrome further, we aimed to both develop and evaluate a method for the post mortem definition of the metabolic syndrome.

**Methods:**

Based on the nationwide Danish SURVIVE study of deceased mentally ill, we established a set of post mortem criteria for each of the harmonized criteria of the metabolic syndrome. We based the post mortem (PM) evaluation on information from the police reports and the data collected at autopsy, such as anthropometric measurements and biochemical and toxicological analyses (PM information). We compared our PM evaluation with the data from the Danish health registries [ante mortem (AM) information, considered the gold standard] from each individual.

**Results:**

The study included 443 deceased individuals (272 male and 171 female) with a mean age of 50.4 (± 15.5) years and a median (interquartile range) post mortem interval of 114 (84–156) hours. We found no significant difference when defining the metabolic syndrome from the PM information in comparison to the AM information (*P *= 0.175). The PM evaluation yielded a high specificity (0.93) and a moderate sensitivity (0.63) with a moderate level of agreement compared to the AM evaluation (Cohen’s κ = 0.51). Neither age nor post mortem interval affected the final results.

**Conclusions:**

Our model of a PM definition of the metabolic syndrome proved reliable when compared to the AM information. We believe that an appropriate estimate of the prevalence of the metabolic syndrome can be established post mortem. However, while neither the PM nor the AM information is exhaustive in terms of defining an individual’s health status, a superlative estimate may be obtained by combining the PM and the AM information. With this model, we open up the possibility of utilising autopsy data for future studies of the metabolic syndrome.

## Background

Individuals with a severe mental illness (SMI), such as schizophrenia, bipolar disorder or major depression, constitute a part of the population that is more prone to suffer from obesity and the related co-morbidities [1–3]. The reasons for this are many-faceted and include socio-economic factors, sedentary lifestyle, diet, smoking and medication [2–4]. It has been demonstrated repeatedly that the segment of the population with SMI suffers from metabolic syndrome (MetS) more frequently in comparison to the background population [1, 5–8].

Metabolic syndrome is comprised of a cluster of reversible risk factors that focus the attention of the clinician to patients with an increased risk of cardiovascular disease (CVD) and type 2 diabetes mellitus (DM). Several definitions of MetS have been proposed by The World Health Organization, The International Diabetes Federation and the National Cholesterol Education Program [9–11]. In 2009, the harmonized criteria of the MetS were proposed including central obesity, raised blood pressure, raised blood glucose, raised triglyceride level (TG), and/or lowered high density lipoprotein (HDL) cholesterol level, of which three have to be met for a person to be diagnosed with the MetS [12]. Individuals suffering from the MetS have double the risk of developing CVD and a five times increased risk of developing DM in comparison to the background population [12]. While some have argued for the dismissal of the MetS as a concept [13, 14], all of the major entities that have defined it recommend further research into it and the factors that dispose to CVD and diabetes [9, 11, 12].

An autopsy is widely considered the ultimate diagnostic tool and several studies have demonstrated the benefit of autopsies in diagnosing overlooked diseases [15–17]. Hereditary heart diseases and the ‘Back to Sleep’ campaign in sudden infant death syndrome are just some of the examples of autopsy based diagnostics and epidemiology of the dead that have prevented morbidity and mortality [18–20].

The aim of this study was to define and evaluate the post mortem (PM) criteria of the MetS, based on the Danish autopsy-based SURVIVE study [21]. Establishing well-defined PM criteria will make it possible to link the morbidity and mortality determined at the autopsy to the MetS and thereby exploit the potential of the autopsy data in elucidating diagnostics, pathophysiology and potential treatment of the MetS. To accomplish this goal, we focused on specific autopsy related data (police reports, biochemical and toxicological results; i.e. PM information) and we tested it against registry data [diagnosis codes and prescribed medication; i.e. ante mortem (AM) information].

## Methods

### Study population

The SURVIVE study [21] included all of the deceased persons, over a 2-year period in Denmark, with a known or suspected psychiatric disease (ICD10 F1–F6, F9) where a forensic autopsy was performed. The deceased were included from May 2013 to May 2015 at all three Departments of Forensic Medicine. The exclusion criteria were: (1) moderate to advanced putrefaction, (2) computed tomography (CT) scan prior to autopsy not performed, and (3) cases of homicide or suspected homicide. We retrieved information from the general practitioner (GP) regarding the deceased’s health status and medication from the police reports concerning the death of each individual. We accessed the Danish National Health Registries and, based on the social security number, retrieved the hospital ICD10 diagnosis codes from 1994 to the time of death and the prescribed medication for each individual.

A total of 500 deceased were included in the SURVIVE study. In accordance with Danish legislation, the next of kin were contacted and consent was obtained in 443 cases (male = 272; female = 171). The ethnicities of the decedents included 435 Caucasians, 5 Africans, 2 Inuit (Greenlanders) and 1 of Middle Eastern origin. We calculated the post mortem interval (PMI) in a similar fashion to Holm and Linnet [22], as half the time from the decedent was last seen alive to he/she was found dead plus the time to autopsy. In cases were death was witnessed, we only used the time from death to autopsy.

The study was approved by the Danish National Committee on Health Research Ethics (Case Number: 1305373) and the National Danish Data Protection Agency (case number: SUND-2016-06).

### Anthropometry and biochemical parameters

Anthropometric measurements were recorded during the forensic autopsy. The height of the deceased was measured in a supine position and the weight was recorded with the decedent naked. The standard waist circumference (WC) was measured halfway between the superior iliac crest and the costal margin [23] and a conversion factor for the WC measurements from the supine to the standing position was applied [24]. The heart was transversally sectioned from the apex to the mid-ventricular level. The left ventricular wall thickness of the anterior, lateral and posterior part was measured at the mid-ventricular level, according to international standards [25].

During the autopsy, when possible, peripheral blood from the internal femoral artery was gathered; if this was not possible, central blood from the heart was sampled. Urine samples were also gathered. A total of 364 blood samples and 248 urine samples were collected; the samples were stored in vials with no additives at − 20 °C or − 80 °C until analysis.

We measured the total cholesterol and triglycerides (TG) using a Reflotron^®^ Plus (Roche, Basel, Switzerland). Glycated haemoglobin (HbA1C) in the blood and the urine albumin/creatinine ratio were measured using a Siemens DCA Vantage^®^ immuno-assay (Siemens, Erlangen, Germany). Standard toxicological analyses of the blood samples were performed, using ultra performance liquid chromatography with tandem mass spectrometry (UPLC MS/MS), to detect the presence of medications related to the MetS (Table 1).

**Table 1:** Specifications for registry searches

Metabolic syndrome criteria	ICD-10 codes	Medication groups	ATC-codes
Hypertriglyceridemia	DE781	Fibrates, Nicotinic acid	C10AB
Hypercholesterolemia	DE780	Statins, Cholestyramine, Cholesterol absorption inhibitors	C10AA, C10AC, C10AX
Hypertension	DI10–DI15	Thiazide, Ca^2+^-antagonists, ACE inhibitors, Angiotensin II receptor blockers, Renin inhibitors, β-blockers, α-blockers, Hydralazine, Centrally active (Moxonidine)	C02AB, C02AC, C02CA, C02DB, C03, C07A, C08, C09A, C09C, C09X
Elevated blood glucose	DE10–DE14	Β-cell stimulants, α-glucosidase inhibitors, Glitazones, DPP-IV-inhibitors, GLP1-analogs, SGLT2-inhibitors	A10B

Clinical diagnoses related to the MetS with the corresponding ICD10 codes, the medication and the ATC-codes used to define the presence and/or treatment of the MetS. The ATC-codes are based on the Danish National Treatment Guidelines

### Registry data

Using the National Danish Health Registry, we searched for diagnosis codes corresponding to each of the MetS criteria except waist circumference. Based on the Danish National Treatment Recommendations [26], we identified whether a deceased had MetS related medication prescribed within the 6 months prior to death (Table 1).

### Post mortem definition of the MetS

We cross-referenced the PM information with the AM information, as shown in Table 2. If just one of the PM criteria in groups 2–5 (Table 2) were met, the corresponding AM criterion was assumed to be fulfilled. A deceased was classified as having the MetS if three or more of the AM criteria were present, according to the harmonized criteria [12]. The cut-off values for total cholesterol, TG, and HbA1C were selected based on the definition of pre-diabetes and the Danish National Treatment Guidelines [27–29]. The urine albumin/creatinine ratio and the left ventricular hypertrophy (LVH) were used as proxy markers for hypertension when underlying kidney disease (ICD10 N0–N2) and cardiomyopathy (ICD10 I42–I43), respectively, had been excluded [24, 25].

**Table 2:** Harmonized criteria for the metabolic syndrome and the corresponding post mortem criteria

	Ante mortem criteria	Post mortem criteria
1	Waist circumference (≥ 94 cm men; ≥ 80 cm women)	Supine waist circumference with conversion factor (≥ 94 cm men; ≥ 80 cm women)
2	Increased TG or treatment hereof	Information from the police report Medication in the PM-toxicology Increased TG in the PM-biochemistry (≥ 1.7 mmol/L)
3	Decreased HDL-cholesterol or treatment hereof	Information from the police report Medication in the PM-toxicology Increased total cholesterol in the PM-biochemistry (≥ 5.0 mmol/L)
4	Hypertension or treatment hereof	Information from the police report Medication in the PM-toxicology Left ventricular wall thickness (≥ 15 mm) Increased urine albumin/creatinine ratio in the PM-biochemistry (> 30 mg/g)
5	Increased fasting blood glucose or treatment hereof	Information from the police report Medication in the PM-toxicology Increased HbA1C in the PM-biochemistry (> 38 mmol/mol)

*HbA1C* glycated haemoglobin, *HDL* high density lipoprotein, *PM* post mortem, *TG* triglyceride

### Statistical analysis

Each of the criteria, except the WC, based on the PM information were compared with the corresponding AM information, as the gold standard. Reproducibility was determined using Cohen’s kappa with agreement assessed according to Landis and Koch [30]. McNemar’s test was used for the contingency tables of the paired samples. To evaluate our PM MetS criteria further, sensitivity, specificity, and the likelihood ratio (LR) were calculated. A Student’s *t* test and a Wilcoxon rank sum test were used for parametric and non-parametric data, respectively. Pearson’s *r* was determined to assess correlations. A *P* value <0.05 was considered significant. All statistical analyses were performed using R v. 3.4.0 [31].

## Results

The mean (± SD) age of the study population was 50.4 (± 15.5) years with men being significantly younger than women (men: 48.0 [± 14.2] years; women: 54.3 [± 16.6] years; *P* < 0.001). We determined a median PMI (interquartile range) of 114 (84–156) hours. We were unable to determine the PMI in 72 cases. All ethnicities fell within the same reference measurement of the WC, as stated by the IDF (≥ 94 cm men; ≥ 80 cm women). The basic anthropometric and biochemical data are listed in Table 3. Adjusting for age in Table 3 did not produce significant differences in either criteria (data not shown). Investigating the effect of PMI on the biochemical measurements, we found a significant correlation with TG (*r *=0.22, *P* < 0.001) but no significant correlations for the other criteria (total cholesterol: *r *=− 0.14, *P* = 0.07. Albumin/creatinine ratio: *r *=0.02, *P *= 0.78. HbA1c: *r *= − 0.01, *P *= 0.82). From the autopsy reports, we concluded that none of the deceased with LVH or an elevated albumin/creatinine ratio suffered from cardiomyopathy or kidney disease.

**Table 3:** Anthropometry and biochemical results related to the metabolic syndrome

Metabolic syndrome criteria	Men	Women	N (male/female)	*P* value
Waist circumference (cm), converted	97.3 (95.3; 99.3)	94.2 (91.3; 97.0)	443 (272/171)	0.08
Triglycerides (mmol/L)^a^	2.90 (2.60; 3.40)	3.00 (2.60; 3.45)	308 (185/123)	0.39
Total cholesterol (mmol/L)^a^	3.00 (< 3.00; 3.20)	3.10 (< 3.00; 3.2)	216 (90/126)	0.11
Albumin/creatinine ratio^b^	16.36 (7.88; 49.91)	22.35 (12.02; 67.59)	248 (75/173)	0.11
Glycated haemoglobin (mmol/mol)^a^	32 (29; 36)	33 (29; 37)	364 (140/224)	0.67

The lower quantification limits for triglycerides was 0.8 mmol/L and it was 3.00 mmol/L for total cholesterol. The upper quantification limit for total cholesterol was 6.8 mmol/L. Not all blood samples could be analysed for triglycerides or cholesterol. The waist circumference is expressed as mean (95% CI). The remaining values are expressed as median (interquartile range). Adjusting for age did not affect the results

^a^Blood samples

^b^Urine samples

We compared our PM definition of the MetS with each of the proposed PM criteria, based on the PM information, with the corresponding AM definition and information. The reproducibility of our combined PM MetS definition resulted in a moderate agreement (κ = 0.51). For the criteria regarding the TG, total cholesterol, hypertension and blood glucose, the agreement ranged from slight to moderate (Table 4). Information on the WC was only available from the PM information and was thus omitted from Tables 4 and 5.

**Table 4:** PM MetS estimation and individual criteria compared to the AM definition and corresponding criteria

Combined MetS definition	Ante Mortem		Cohen’s κ (male/female)
	MetS yes (male/female)	MetS no (male/female)	
Post mortem			
MetS yes	29 (16/13)	27 (14/13)	0.51 (0.56/0.45)
MetS no	17 (7/10)	370 (235/135)	

Individual criteria		Criterion met	Criterion not met	Cohen's κ (male/female)
Post mortem				
Triglycerides		NA	NA	0.19 (NA/NA)
Total cholesterol	Criterion met	18 (13/5)	7 (NA)	0.36 (0.47/0.22)
	Criterion not met	44 (21/23)	374 (234/140)	
Hypertension	Criterion met	103 (55/48)	153 (106/47)	0.24 (0.19/0.33)
	Criterion not met	26 (14/12)	161 (97/64)	
Blood glucose	Criterion met	34 (18/16)	40 (21/19)	0.55 (0.54/0.56)
	Criterion not met	5 (NA)	364 (229/135)	

Due to restrictions from statistics Denmark regarding the disclosure of aggregated microdata (N ≤ 3), the table regarding the triglyceride criterion has been omitted and NAs used in appropriate fields in the other criteria

*MetS* metabolic syndrome

**Table 5:** Evaluation of the PM definition of the metabolic syndrome and individual criteria

	Sensitivity (male/female)	Specificity (male/female)	Likelihood ratio (95% CI)	Difference PM vs. AM (95% CI)	*P* value
PM definition MetS	0.63 (0.70/0.57)	0.93 (0.94/0.91)	9.27 (6.05; 14.19)	2.3% (− 0.7%; 5.2%)	0.175
PM cholesterol	0.29 (0.38/0.17)	0.98 (0.98/0.98)	15.80 (6.89; 36.27)	− 8.4% (− 11.4%; − 5.3%)	< 0.001
PM hypertension	0.80 (0.80/0.80)	0.51 (0.48/0.58)	1.64 (1.42; 1.89)	34.5% (29.3%; 39.8%)	< 0.001
PM triglycerides	1 (NA/NA)	0.30 (NA/NA)	1.42 (1.34; 1.51)	70.0% (65.7%; 74.2%)	< 0.001
PM triglycerides w/o PM biochemistry	0.50 (NA/NA)	0.98 (NA/NA)	31.50 (6.65; 151.21)	1.4% (0.1%; 2.6%)	0.077
PM glucose	0.87 (0.82/0.94)	0.90 (0.92/0.88)	8.81 (6.41; 12.10)	7.9% (5.0; 10.8%)	< 0.001

All results are evaluated comparing the PM definition or criterion to the AM definition and the corresponding criterion. Gender-specific sensitivity and specificity for triglycerides was not calculated due to restrictions from Statistics Denmark regarding disclosure of aggregated microdata (N < 3)

*AM* ante mortem, *CI* confidence interval, *MetS* metabolic syndrome, *PM* post mortem, *w/o* without

We found no significant difference when we estimated the MetS based only on the PM information, in comparison to estimating the MetS based only on the AM information (Table 5). Our PM definition resulted in high specificity (0.93) and moderate sensitivity (0.63) with a reasonable likelihood ratio (LR) (9.27) (Table 5).

The biochemical measurements of the PM TG were high when compared to the cut-off value (Table 3). Although using the PM triglyceride biochemistry results for the triglyceride criteria produced no false negative results, hence the perfect sensitivity, this yielded an immense overestimation. Omitting the PM triglyceride biochemistry results produced a far more accurate estimation, with no significant difference, between the PM and the AM information. None of the deceased had a total cholesterol level above 4.0 mmol/L in the PM biochemical analyses. Therefore, using only the PM information for the cholesterol criteria resulted in a significant underestimation of that criterion. Conversely, using only the PM information on the hypertension and glucose criteria significantly overestimated those criteria.

## Discussion

To our knowledge, this is the first study that defined the criteria for the diagnosis of the MetS in a post mortem perspective.

We opted to use the AM information from the registries as the gold standard. A priori, the specificity of this information is close to one. The registry information is, however, not exhaustive regarding an individual’s health status. Only diagnoses from hospital admissions were recorded; diagnoses from the GPs of the individual subjects were not available through our registries. Conversely, in a forensic autopsy setting, access is available to the police reports, which include the police questioning of the GPs regarding a decedent’s health status. Although this information might not be exhaustive, it may be regarded as information with a high specificity. The registry information regarding prescribed medication reflects the diagnoses that a GP finds appropriate to treat and is consequently an indirect source for measuring a person’s health status. A caveat when defining the health status of individuals with SMI from registries or GPs is that the prevalence of dyslipidaemia and hypertension are frequently underestimated and undertreated [32]. Although our proposed PM definition of the MetS was not significantly different from the AM definition, it yielded only a moderate level of agreement. While there were some gender differences in Cohen’s κ for some of the individual criteria, the gender-specific agreement in the final PM model was quite similar. Since the prevalence of the MetS in our study cohort is relatively low, the calculated Cohen’s κ might underestimate the actual agreement between the PM and the AM definition and criteria [33]. Therefore, the sensitivity and specificity may reflect the most correct estimate of agreement. Again, no gender differences regarding sensitivity and specificity were evident. In addition, since the information originates from different sources in our setup, comparing the PM information with the AM information will generally produce some discrepancies. Consequently, one should regard the level of agreement with some reservations.

Measuring and interpreting the PM biochemical analyses posed several challenges. The total cholesterol decreases PM leaving only elevated concentrations interpretable [34, 35]. Since none of our subjects had a total cholesterol above 4.0 mmol/L, our findings may corroborate the challenge of analysing cholesterol in PM samples. The levels of PM TG have been reported to be affected by too many variables to make it useful for interpretation [34, 35]. In line with the study by Girard et al. [35], the measured TG in our study increased with PMI. Moreover, the reference values of cholesterol and TG in the living are frequently based on a premise of fasting. We concluded that total cholesterol and TG were imprecise parameters and omitted them from our final MetS estimation. While direct measurement of blood glucose levels PM is not feasible [36], several studies have proven HbA1C as a robust marker of long-term blood glucose status in PM measurements [34, 36–40]. As we used an immune-assay to measure HbA1c, our results might be biased towards higher values due to diminished specificity compared to chromatographic measurements [36]. Our PM information differed significantly from the AM information. Although this could be a result of underreported and undertreated hyperglycaemia in people with SMI [32], it may reflect the fact that our cut off value of 38 mmol/mol does not yield an ICD10 code of diabetes in a clinical setting; contributing to our PM definition overestimating the prevalence of this criterion.

Diagnosing hypertension at autopsy can be problematic. Few markers exist and they rely on the exclusion of other diseases. In hypertension, the myocytes in the left ventricle of the heart undergo hypertrophy. Although it manifests most frequently as concentric hypertrophy, it can also present as eccentric hypertrophy. While other diseases may affect the pattern of hypertrophic response, hypertension is still a part of these conditions [41]. When it comes to ruling out cardiomyopathies, autopsy data, specifically histology, provides one of the hallmarks of diagnosing these diseases. Therefore, we considered LVH, excluding cardiomyopathies, as an appropriate marker for hypertension.

The WC was the only criterion that we were unable to validate with AM information. However, as one of the exclusion criteria was moderate to advanced putrefaction, cases with markedly PM bloating of the abdomen were not included. In addition, the use of a conversion factor from the supine to the standing position [24] further corrected the estimated WC.

Selection bias must be considered since the study is based on forensic autopsy material. A forensic autopsy is performed on request from the police in cases of accidents, suicides, (suspected) homicides and when the manner and/or cause of death remains unknown at the medico-legal examination. Therefore, the results are in line with most other study populations based on forensic autopsies that are descriptive for a specific study population and not necessarily representative for the general population. In such a study population, a precise setup of inclusion criteria—such as age, gender, specific diagnosis and equivalent healthy controls—may accommodate some of the selection bias. Furthermore, as the present study was based on a study population of deceased individuals with SMI, we cannot rule out the possibility of a different result if the PM model was employed on deceased mentally healthy individuals. However, with the aforementioned underestimation and undertreatment of dyslipidaemia in individuals with SMI [32], the evaluation of the PM model based on individuals with SMI might actually underestimate the specificity of the model compared to the same evaluation based on a mentally healthy study population.

## Conclusions

With this study, we proposed a valid estimate for the PM diagnosis of the MetS. While neither the PM nor the AM information are exhaustive regarding an individual’s health status, combining both the PM and the AM information might provide the best estimation. However, bearing in mind that the result is an approximation and not a conclusive answer, employing PM information alone is applicable. Although PM TG and total cholesterol measurements are unreliable and should be omitted, PM HbA1C remains a valid measurement. Employing autopsy material and our PM MetS definition, we believe that autopsy-based data may promote the research of the MetS and associated morbidities from a novel perspective.

## Abbreviations

AM: ante mortem
ATC: Anatomical Therapeutic Chemical Classification System
CI: confidence interval
CT: computed tomography
CVD: cardiovascular disease
DM: diabetes mellitus
GP: general practitioner
HbA1c: glycated haemoglobin
HDL: high-density lipoprotein
ICD: international classification of diseases
UPLC MS/MS: ultra performance liquid chromatography with tandem mass spectrometry
LR: likelihood ratio
LVH: left ventricular hypertrophy
MetS: metabolic syndrome
PM: post mortem
PMI: post mortem interval
SMI: severe mental illness
TG: triglyceride
WC: waist circumference
w/o: without

## References

[1] Van Gaal LF (2006). Long-term health considerations in schizophrenia: metabolic effects and the role of abdominal adiposity. Eur Neuropsychopharmacol.

[2] Nørgaard HCB, Birk M, Jakobsen AS, Speyer H (2016). Overweight and obesity in patients with severe mental illness. Ugeskr Laeger.

[3] Avila C, Holloway AC, Hahn MK, Morrison KM, Restivo M, Anglin R (2015). An overview of links between obesity and mental health. Curr Obes Rep.

[4] De Hert M, Dekker JM, Wood D, Kahl KG, Holt RIG, Möller H-J (2009). Cardiovascular disease and diabetes in people with severe mental illness position statement from the European Psychiatric Association (EPA), supported by the European Association for the Study of Diabetes (EASD) and the European Society of Cardiology (ESC). Eur Psychiatry.

[5] Vancampfort D, Stubbs B, Mitchell AJ, De Hert M, Wampers M, Ward PB (2015). Risk of metabolic syndrome and its components in people with schizophrenia and related psychotic disorders, bipolar disorder and major depressive disorder: a systematic review and meta-analysis. World Psychiatry.

[6] Vancampfort D, Correll CU, Wampers M, Sienaert P, Mitchell AJ, De Herdt A (2014). Metabolic syndrome and metabolic abnormalities in patients with major depressive disorder: a meta-analysis of prevalences and moderating variables. Psychol Med.

[7] Vancampfort D, Vansteelandt K, Correll CU, Mitchell AJ, De Herdt A, Sienaert P (2013). Metabolic syndrome and metabolic abnormalities in bipolar disorder: a meta-analysis of prevalence rates and moderators. Am J Psychiatry.

[8] Mitchell AJ, Vancampfort D, Sweers K, van Winkel R, Yu W, De Hert M (2013). Prevalence of metabolic syndrome and metabolic abnormalities in schizophrenia and related disorders—a systematic review and meta-analysis. Schizophr Bull.

[9] Expert Panel on Detection, Evaluation, and Treatment of High Blood Cholesterol in Adults (2001). Executive summary of the third report of the national cholesterol education program (NCEP) expert panel on detection, evaluation, and treatment of high blood cholesterol in adults (adult treatment panel III). JAMA.

[10] Alberti KGMM, Zimmet P, Shaw J (2005). The metabolic syndrome—a new worldwide definition. Lancet.

[11] Alberti KGMM, Zimmet PZ. Definition, diagnosis and classification of diabetes mellitus and its complications. WHO; 1999. http://apps.who.int/iris/bitstream/10665/66040/1/WHO_NCD_NCS_99.2.pdf. Accessed 10 July 2017.

[12] Alberti KGMM, Eckel RH, Grundy SM, Zimmet PZ, Cleeman JI, Donato KA (2009). Harmonizing the metabolic syndrome: a joint interim statement of the International Diabetes Federation Task Force on Epidemiology and Prevention; National Heart, Lung, and Blood Institute; American Heart Association; World Heart Federation; International Atherosclerosis Society; and International Association for the Study of Obesity. Circulation.

[13] Kahn R (2008). Metabolic syndrome—what is the clinical usefulness?. Lancet.

[14] Sattar N, McConnachie A, Shaper AG, Blauw GJ, Buckley BM, de Craen AJ (2008). Can metabolic syndrome usefully predict cardiovascular disease and diabetes? Outcome data from two prospective studies. Lancet Lond Engl.

[15] Shojania KG, Burton EC, McDonald KM, Goldman L (2003). Changes in rates of autopsy-detected diagnostic errors over time: a systematic review. JAMA.

[16] Shojania KG, Burton EC (2008). The vanishing nonforensic autopsy. N Engl J Med.

[17] Thurnheer R, Hoess C, Doenecke C, Moll C, Muntwyler J, Krause M (2009). Diagnostic performance in a primary referral hospital assessed by autopsy: evolution over a ten-year period. Eur J Intern Med.

[18] Willinger M, Hoffman HJ, Hartford RB (1994). Infant sleep position and risk for sudden infant death syndrome: report of meeting held January 13 and 14, 1994, National Institutes of Health, Bethesda. MD. Pediatrics.

[19] American Academy of Pediatrics Task Force on Sudden (2005). Infant Death Syndrome. The changing concept of sudden infant death syndrome: diagnostic coding shifts, controversies regarding the sleeping environment, and new variables to consider in reducing risk. Pediatrics.

[20] Napolitano C, Bloise R, Monteforte N, Priori SG (2012). Sudden cardiac death and genetic ion channelopathies: long QT, Brugada, short QT, catecholaminergic polymorphic ventricular tachycardia, and idiopathic ventricular fibrillation. Circulation.

[21] SURVIVE—let the dead help the living. http://retsmedicin.ku.dk/english/research/surviveprojects. Accessed 29 Jan 2018.

[22] Holm KMD, Linnet K (2015). Distribution of enantiomers of methadone and its main metabolite EDDP in human tissues and blood of postmortem cases. J Forensic Sci.

[23] World Health Organization (2011). Waist circumference and waist-hip ratio: report of a WHO expert consultation, Geneva, 8–11 December 2008.

[24] Waninge A, Ligthart KAM, Kramer J, Hoeve S, van der Schans CP, Haisma HH (2010). Measuring waist circumference in disabled adults. Res Dev Disabil.

[25] Basso C, Aguilera B, Banner J, Cohle S, d’Amati G, de Gouveia RH (2017). Guidelines for autopsy investigation of sudden cardiac death: 2017 update from the Association for European Cardiovascular Pathology. Virchows Arch.

[26] Lægehåndbogen https://www.sundhed.dk/sundhedsfaglig/laegehaandbogen/. Accessed 26 Jul 2016. **(Danish)**.

[27] American Diabetes Association (2014). Diagnosis and classification of diabetes mellitus. Diabetes Care.

[28] Lægehåndbogen—Total cholesterol. https://www.sundhed.dk/sundhedsfaglig/laegehaandbogen/undersoegelser-og-proever/klinisk-biokemi/blodproever/kolesterol-total/. Accessed 26 Jul 2016. **(Danish)**.

[29] Lægehåndbogen—Hypertriglycerdaemi. https://www.sundhed.dk/sundhedsfaglig/laegehaandbogen/endokrinologi/tilstande-og-sygdomme/lipidforstyrrelser/hypertriglyceridaemi/. Accessed 26 Jul 2016. **(Danish)**.

[30] Landis JR, Koch GG (1977). The measurement of observer agreement for categorical data. Biometrics.

[31] R Core Team. R: a language and environment for statistical computing. Vienna: R Foundation for Statistical Computing. https://www.R-project.org/. Accessed 18 Apr 2017.

[32] Nasrallah HA, Meyer JM, Goff DC, McEvoy JP, Davis SM, Stroup TS (2006). Low rates of treatment for hypertension, dyslipidemia and diabetes in schizophrenia: data from the CATIE schizophrenia trial sample at baseline. Schizophr Res.

[33] Hoehler FK (2000). Bias and prevalence effects on kappa viewed in terms of sensitivity and specificity. J Clin Epidemiol.

[34] Uemura K, Shintani-Ishida K, Saka K, Nakajima M, Ikegaya H, Kikuchi Y (2008). Biochemical blood markers and sampling sites in forensic autopsy. J Forensic Leg Med.

[35] Girard C, Scarpelli MP, Tettamanti C, Palmiere C (2017). Postmortem evaluation of cholesterol, triglyceride, and apolipoprotein levels. Int J Legal Med.

[36] Hess C, Musshoff F, Madea B (2011). Disorders of glucose metabolism—post mortem analyses in forensic cases: part I. Int J Legal Med.

[37] Chen J-H, Michiue T, Inamori-Kawamoto O, Ikeda S, Ishikawa T, Maeda H (2015). Comprehensive investigation of postmortem glucose levels in blood and body fluids with regard to the cause of death in forensic autopsy cases. Leg Med.

[38] Madea B, Musshoff F (2007). Postmortem biochemistry. Forensic Sci Int.

[39] Belsey SL, Flanagan RJ (2016). Postmortem biochemistry: current applications. J Forensic Leg Med.

[40] Chen C, Glagov S, Mako M, Rochman H, Rubenstein AH (1983). Post-mortem glycosylated hemoglobin (HbA1c): evidence for a history of diabetes mellitus. Ann Clin Lab Sci.

[41] Drazner MH (2011). The progression of hypertensive heart disease. Circulation.

